# Alcohol Consumption, Bone Mineral Density, and Risk of Osteoporotic Fractures: A Dose–Response Meta-Analysis

**DOI:** 10.3390/ijerph19031515

**Published:** 2022-01-28

**Authors:** Justyna Godos, Francesca Giampieri, Emanuele Chisari, Agnieszka Micek, Nadia Paladino, Tamara Y. Forbes-Hernández, José L. Quiles, Maurizio Battino, Sandro La Vignera, Giuseppe Musumeci, Giuseppe Grosso

**Affiliations:** 1 Department of Biomedical and Biotechnological Sciences, University of Catania, 95123 Catania, Italy; justyna.godos@gmail.com (J.G.); nadia.paladino97@gmail.com (N.P.); g.musumeci@unict.it (G.M.); 2 Research Group on Food, Nutritional Biochemistry and Health, Universidad Europea del Atlántico, 39011 Santander, Spain; f.giampieri@univpm.it (F.G.); jlquiles@ugr.es (J.L.Q.); 3 Rothman Orthopaedic Institute, Thomas Jefferson University, Philadelphia, PA 19107, USA; emanuele.chisari@rothmanortho.com; 4 Institute of Nursing and Midwifery, Faculty of Health Sciences, Medical College, Jagiellonian University, 31-501 Krakow, Poland; agnieszka.micek@uj.edu.pl; 5 Department of Physiology, Institute of Nutrition and Food Technology ‘‘José Mataix”, Biomedical Research Centre, University of Granada, 18100 Granada, Spain; tforbes@uvigo.es; 6 Department of Clinical Sciences, Polytechnic University of Marche, 60131 Ancona, Italy; m.a.battino@univpm.it; 7 International Joint Research Laboratory of Intelligent Agriculture and Agri-Products Processing, Jiangsu University, Zhenjiang 212013, China; 8 Department of Clinical and Experimental Medicine, University of Catania, 95131 Catania, Italy; sandrolavignera@unict.it; 9 Research Center on Motor Activities (CRAM), University of Catania, 95123 Catania, Italy

**Keywords:** alcohol, osteoporosis, bone mineral density, meta-analysis, bone health, fractures

## Abstract

Excess alcohol consumption is known to be detrimental to human health. However, the role of light-to-moderate alcohol intake is under investigation for potential certain health benefits—mostly related to the cardiovascular system. Nevertheless, there is no univocal agreement on this matter, and research is still ongoing to clarify whether there might be other potential outcomes affected by alcohol intake. In this regard, there is evidence that excess alcohol intake may negatively influence the risk of osteoporotic fractures. However, there is no comprehensive evidence of literature assessing the role of alcohol consumption in bone mineral density (BMD) and the risk of osteoporotic fractures. Thus, the aim of this study was to quantitatively assess the dose–response relationship between alcohol intake and BMD and risk of osteoporotic fractures. The Embase and MEDLINE electronic databases were searched from their inception to December 2021 for articles providing a quantifiable measurement of alcohol consumption for at least three categories and (1) a measurement of BMD (and dispersion as continuous variables) in some area of the body or (2) risk of osteoporotic fracture provided as relative risk (RR) or hazard ratio (HR), with a 95% confidence interval (CI) as the measure of the association of each category with alcohol intake. A total of 11 studies including 46,916 individuals with BMD assessment and 8 studies including 240,871 individuals with risk of fracture analysis were included. Compared to non-drinkers, consumption of up to two standard drinks of alcohol per day was correlated with higher lumbar and femur neck BMD values, while up to one standard drink of alcohol was correlated with higher hip BMD compared to no alcohol consumption. Higher risk of hip fractures was found starting from three standard drinks of alcohol per day (RR = 1.33, 95% CI: 1.04; 1.69 for three alcoholic drinks/d, and RR = 1.59, 95% CI: 1.23; 2.05 for four alcoholic drinks/d) compared to no alcohol consumption, with no evidence of heterogeneity. Concerning the risk of any osteoporotic fractures, the risk steadily increased with higher intake of alcohol, although never reaching statistical significance. In conclusion, there is consistent evidence that increased alcohol consumption is associated with higher risk of osteoporotic hip fracture; however, the role of alcohol at lower doses is uncertain, as BMD was even higher in light drinkers compared to abstainers.

## 1. Introduction

The extension of lifespan and the increasing aging population are changing the global burden of diseases, with age-related conditions becoming increasingly prevalent among the older population [1]. Bone health is one of the major interests in aging research, and is an important target to reduce the risk of fractures and increase quality of life in older individuals [2]. Osteoporosis represents an important cause of fracture in this population, affecting more than 200 million people worldwide, and resulting in more than 5 million disability-adjusted life years (DALYs) globally per year [3]. Osteoporosis is a condition characterized by loss of bone mass and deterioration of bone microarchitecture [4]. Using standard deviation scores of bone mineral density (BMD) related to peak bone mass in healthy young women, osteoporosis is defined as a BMD T score of −2.5 SD or less, and low bone mass (osteopenia) as a BMD T-score between −1 and −2.5 SD [5]. The only validated method to recognize the disease is dual-energy X-ray absorptiometry (DEXA), which can be an expensive tool [4]. The occurrence of osteoporosis increases the risk of bone fracture, substantially decreasing the quality of life of older individuals [6]. Thus, it is imperative to investigate potential risk factors for osteoporosis in order to prevent fractures.

Most interventions focusing on bone health aim to decrease the risk of falls and osteoporosis in the general population, with only a select subpopulation undergoing targeted screening and treatment [7,8]. Lifestyle interventions have been investigated for potential roles in the occurrence of osteoporosis—healthy dietary practices, regular exercise, and smoking cessation are in fact widely considered the gold standard to decrease disease burden [9]. Among nutritional factors to be taken into consideration, vitamin D and calcium have been considered to be among the most important factors to investigate, given their role in bone health, although the evidence from clinical trials of supplementation is limited [10]. In contrast, the role of the overall diet [11] and other food groups—including dairy, fruit, and vegetable intake—has emerged as another potential factor to be considered for bone health [12]. Moreover, several nutrients—including omega-3 fatty acids, magnesium, potassium, vitamins A, C, E, and K, and carotenoids—have been shown to be more important than previously thought [13]. Among factors that may exert negative effects on BMD, alcohol consumption has long been studied for being one of the most detrimental [14]. However, while excess alcohol consumption has been widely associated with increased risk of developing osteoporosis, there are still controversial data regarding light-to-moderate alcohol intake, with no univocal agreement in existing studies [15]. In light of the common consumption of alcohol and its potential influence on bone health, we aimed to systematically investigate the relationship between BMD as a proxy of bone health and alcohol consumption, and to test the risk of fracture associated with the variation in alcohol consumption.

## 2. Materials and Methods

### 2.1. Search Strategy and Study Selection

The meta-analysis was performed in accordance with the Preferred Reporting Items for Systematic Reviews and Meta-Analyses (PRISMA) guidelines [16].

Two authors (J.G. and G.G.) separately screened the Embase and MEDLINE databases to search from their inception to December 2021 for articles describing the association between alcohol consumption and BMD or the risk of osteoporotic fractures. In the process of exploring the literature, the following search terms including terms on alcohol consumption, osteoporotic fracture, BMD measurements, and study design were adapted: (alcoholic beverages OR alcohol OR beer OR wine) AND (drinking OR intake OR consumption OR alcohol abuse) AND (osteoporosis OR Bone disease metabolic OR BMD OR Bone Mineral Density). Only English language studies were considered for inclusion. To establish eligible studies, the investigators first screened the titles and abstracts, and then read the full texts of the retrieved articles. Finally, potentially missed records were identified via examination of the references of previously selected positions.

Concerning the variable of exposure, the studies were included if they provided a quantifiable measure of alcohol consumption for at least three categories. Concerning the outcomes, the included studies had to satisfy the following inclusion criteria: (1) provided a measurement of BMD (and dispersion as continuous variables) in some area of the body in relation to alcohol consumption; or (2) provided incidence risk of osteoporotic fracture given as relative risk (RR) or hazard ratio (HR), with a 95% confidence interval (CI) as the measure of the association for dichotomous outcomes for each category of exposure. Given the measure of risk to be included in the meta-analysis, only studies with a prospective design were included when considering the risk of osteoporotic fracture meta-analysis. Intervention trials and non-original research—such as reviews, editorials, and commentaries—were not considered for inclusion. Effect sizes presented for the same population in more than one article were deemed as duplicates and only the largest number of cases/participants or the study with the longest follow-up for the endpoint of interest was included. In the case of reports that estimated results for both genders together and separately for men and women, data for individual sex was used in the analysis.

### 2.2. Data Extraction

Based on a standardized table with predefined headings, two authors (J.G. and G.G.) independently performed data extraction. The abstracted information from the included studies comprised (1) first author name, (2) year of publication, (3) country, (4) design, (5) sample size, (6) sex and mean age of participants, and (7) follow-up (for prospective cohort studies). Additionally, some data were extracted for each category of alcohol intake, including (1) number of individuals, (2) average alcohol intake, (3) mean and standard deviation of BMD for different individual locations, (4) relative measure of incidence of osteoporotic fracture, and (5) characteristics of selected confounders. Inconsistencies were discussed to reach a decision by consensus. The quality of the included studies was assessed using the Newcastle–Ottawa Quality Assessment Scale (NOS), which scores three domains: selection (4 points), comparability (2 points), and outcome (3 points).

### 2.3. Statistical Analysis

The mean BMD and standard deviation or the RR/HR of fracture were assigned to each category of alcohol consumption. All statistics were extracted from the most fully adjusted models. Meta-analyses of differences in means were fitted separately for lumbar, femur neck, and hip BMD measurements. To combine aggregated data on the differences in means, a flexible method of dose–response meta-analysis was applied [17]. First, in each study, the mean BMD measurement for the group of non-consumers of alcohol was subtracted from the mean BMD measurement assigned to consecutive doses of alcohol. Calculated study-specific mean differences (MDs), being based on common referent values, were not independent; thus, the covariance between them was calculated and then used together with accompanying effect sizes to determine the study-specific dose–response curves. Finally, the estimated intra-study coefficients were combined by means of multivariate meta-analysis. For testing the association of alcohol consumption with the risk of hip and any osteoporotic fractures, the meta-analysis of extreme categories of alcohol consumption (highest versus lowest) and nonlinear dose–response meta-analysis were performed on the log-transformed HRs/RRs. The shape of all curves was modelled using restricted cubic splines with three knots at fixed percentiles of the alcohol consumption distribution (10%, 50%, and 90%). The variance–covariance matrices of the sampling errors were extracted, and the results of individual cohorts reflecting dose–response associations were combined in the two-stage multivariate meta-analysis, taking into account the correlations between the parameters within each study [18,19]. The role of potential confounders was tested using bivariate meta-analysis. Firstly, the intercepts and slopes of the linear regression examining the relationships between alcohol consumption and age, BMI, and percentages of current smokers and physically inactive individuals were estimated in specific cohorts. Next, vectors of retrieved coefficients were meta-analyzed between studies [20]. Random effects models were applied in all analyses. Heterogeneity was evaluated by the I^2^ statistic—with cutoff points of <25%, 25–50%, 50–75%, and >75%, corresponding to no, small, moderate, and significant heterogeneity, respectively—and by Cochran’s Q test, with *p*-value of < 0.1 regarded as an indicator of significant inter-study heterogeneity. Publication bias was evaluated by a visual inspection of funnel plots, and was complemented by the formal regression test. Stratified analysis was performed for sex and geographical region. All analyses were performed with R software version 4.0.2 (Development Core Team, Vienna, Austria), and significance was set at a level of 0.05.

## 3. Results

### 3.1. Study Selection and Main Characteristics

**Figure 1:**
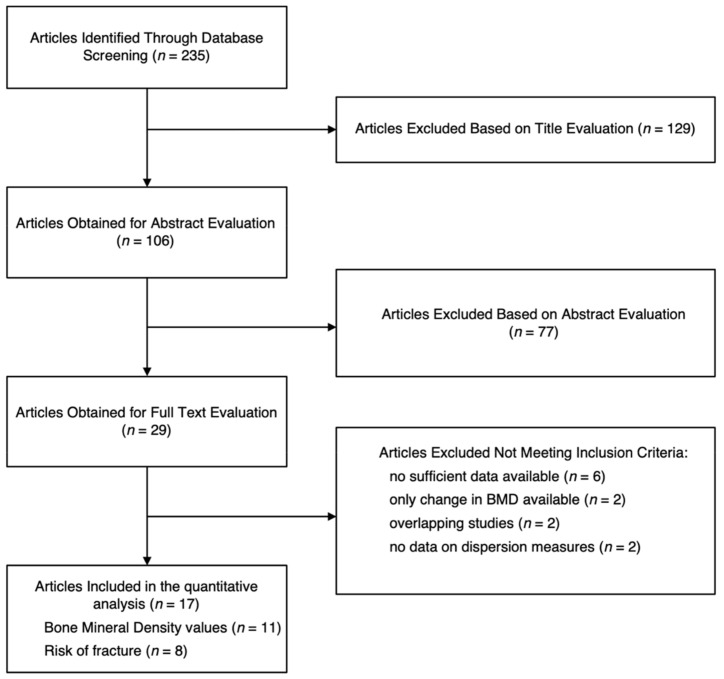
Flowchart of the study selection process.

Out of 235 articles resulting from the search, 106 were examined in detail by their abstract, and 29 by their full text. After excluding 12 papers for not meeting the inclusion criteria, the remaining studies (*n* = 17) were included for the quantitative analyses—11 studies [21,22,23,24,25,26,27,28,29,30,31] providing data on BMD measurements and 8 studies [24,25,32,33,34,35,36,37] providing risk of fracture (Figure 1).

The main characteristics of the studies included are presented in Table 1. Out of 17 studies, 10 were conducted in the US, 3 in Europe, 2 in Asia, 1 in Australia, and 1 was a multicenter study, including an overall number of 275,927 patients. The mean age ranged from 48 to 79 years old. A total of 7 studies provided measures for hip, 8 for spine, and 11 for femur (neck) BMD. The studies providing BMD measurements included 46,916 patients; the studies providing risk of fracture included 240,871 individuals and 8014 fractures. Alcohol consumption was generally assessed through direct questions included in food frequency questionnaires (FFQs). The overall quality of the studies was high (data not shown).

### 3.2. Alcohol Consumption and BMD

**Figure 2:**
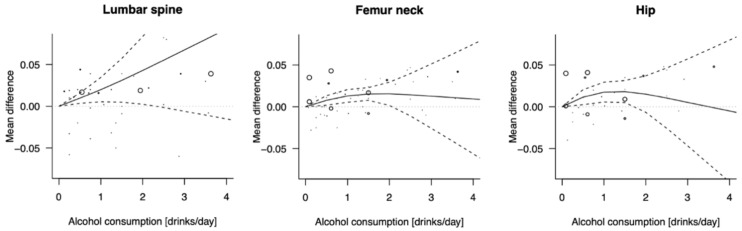
Graphical representation of summary mean differences of bone mineral density in different body segments between various doses of alcohol consumption vs. no consumption.

The relationship between daily alcohol consumption and BMD measures is shown in Figure 2, while numerical values are reported in Table S1. Compared to non-drinkers, consumption of up to two standard drinks of alcohol per day was related to higher lumbar (MD = 0.043, 95% CI: 0.002; 0.083) and femur neck (MD = 0.016, 95% CI: 0.002; 0.030) BMD values; moreover, there was also an increase associated with one standard drink of alcohol compared to no alcohol consumption for hip BMD (MD = 0.018, 95% CI: 0.006; 0.030). However, for higher intakes of alcohol there was no substantial change in BMD values (Figure 2).

The role of potential confounders was investigated, and the results are reported in Table S2 and Figure S1. Age and prevalence of physical inactivity were inversely associated with alcohol consumption, while BMI and current smokers were not associated; when the analyses were carried out by sex, both age and BMI were associated with alcohol consumption, albeit the former inversely and the latter directly.

Subgroup analyses by sex and geographical area are reported in Tables S3 and S4, respectively. There was a potential effect modification by sex, as the findings reported for lumbar BMD were significant in all categories of exposure when considering datasets from men, but not from women; moreover, the differences in femur neck and hip BMD between one standard drink and no alcohol consumption were significant when stratified by sex (Table S3). In terms of the geographical region, lumbar and femur neck BMD values were consistently increased in relation to alcohol intake in non-US studies, while femur neck and hip BMD values were increased for one standard drink of alcohol in studies conducted in the US (Table S4).

### 3.3. Alcohol Consumption and Risk of Fracture

**Figure 3:**
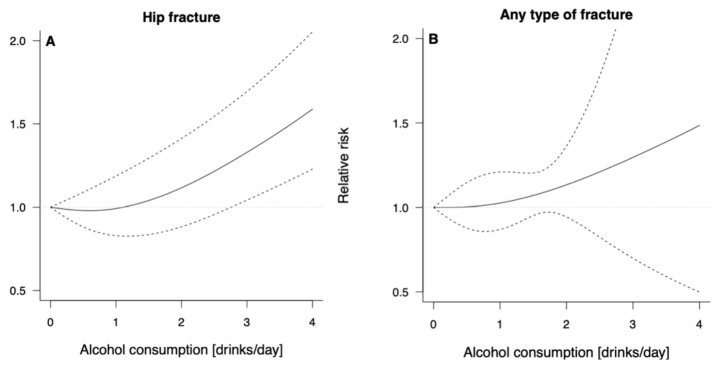
Graphical representation of the dose–response meta-analysis of the risk of hip and any fracture for various doses of alcohol consumption.

The dose–response association between alcohol consumption and risk of hip and any osteoporotic fractures is graphically presented in Figure 3; numerical data are reported in Table 2. Higher risk of hip fractures was found starting from three standard drinks of alcohol per day (RR = 1.33, 95% CI: 1.04; 1.69 for three alcoholic drinks/d, and RR = 1.59, 95% CI: 1.23; 2.05 for four alcoholic drinks/d) compared to no alcohol consumption, with no evidence of heterogeneity. Concerning the risk of any osteoporotic fractures, the risk steadily increased with higher intake of alcohol, although never reaching statistical significance.

**Figure 4:**
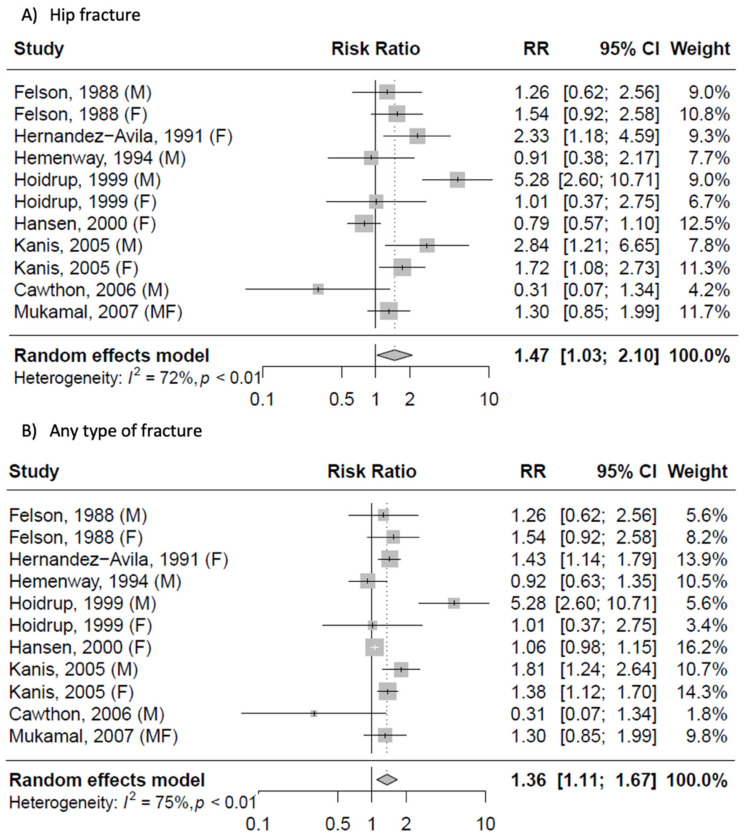
Meta-analysis of the risk of hip and any fracture for the highest vs. lowest categories of alcohol consumption.

The analysis of risk of fracture when comparing extreme categories of alcohol consumption is presented in Figure 4; both analyses showed an increased risk of hip fracture (RR = 1.47, 95% CI: 1.03, 2.10) and any osteoporotic fractures (RR = 1.36, 95% CI: 1.11, 1.67), with evidence of heterogeneity (I^2^ = 72%, *p* < 0.01 and I^2^ = 75%, *p* < 0.01, respectively) and no evidence of publication bias (*p* = 0.464 and *p* = 0.177; Figure S2). The subgroup analysis by sex showed no differences between men and women for hip fractures, but a significant increase in the risk of any fractures only in women (Figure S3). The subgroup analysis by geographical region revealed that the association between high alcohol consumption and risk of hip and any fractures was significant in studies conducted outside the US (Figure S4); however, lack of significance among US samples depended on the results of one study, after the exclusion of which the risk estimates were significant.

## 4. Discussion

In this study, we summarized existing evidence of the association between alcohol consumption and osteoporosis, testing the relationship with BMD and risk of osteoporotic fractures. The analyses led to congruent results, suggesting null relationship between moderate alcohol intake (cautiously quantified as one standard drink per day) and BMD, with a steadily increased risk of fracture for higher alcohol consumption.

The vast majority of scientific literature agrees that excessive alcohol consumption is substantially detrimental to human health [38]. Several studies have provided a global perspective on this matter, emphasizing the proportion of the health burden related to alcohol as one of the major behavioral risk factors for health [39]. However, moderate alcohol consumption is a characteristic feature of certain dietary patterns, such as the Mediterranean diet, and represents a bond with the cultural and religious heritage of certain populations, which has been associated with certain health benefits [40]. Moreover, another feature related to moderate alcohol intake is characterized by its typical consumption during meals, which represents a crucial difference from modern patterns of alcohol consumption characterized by binge drinking in different contexts than meals [41,42,43]. Although the early paradigm associated with the Mediterranean diet identified moderate alcohol consumption, with red wine as an alcohol source, a growing body of literature shows that the relationship between alcoholic beverages and mortality is rather consistent for both wine and beer, resulting in reduced risk in moderate consumers compared to abstainers or heavy drinkers [44]. Moreover, the results from prospective cohorts suggest that moderate alcohol consumption is inversely associated with risk of hip fractures in middle-aged and older men and women [45]. It should be noted that, in addition to the alcoholic content, these beverages are characterized by a variety of bioactive compounds—mainly polyphenols—which may explain, at least in part, the potential benefits for health [46]. It is not surprising that there is no unanimous agreement on the role of alcoholic beverages in human health, especially considering the uncertain role of moderate alcohol consumption [47].

There are several mechanisms providing the rationale for the detrimental effects of excessive alcohol intake on bone health. Moreover, chronic alcohol consumption has been associated with alteration in bone remodeling, whereby bone formation is generally uncoupled with bone resorption [48]. Specifically, alcohol seems to impair the bone microarchitecture, affecting both cortical thickness and trabecular bone volume [49]. These effects may depend on the inhibition of osteoblastic differentiation, proliferation, and activity, which are crucial to the building process, and also contribute to the repair of deficient bone [50]. Other indirect mechanisms underlying these effects may include hormone dysregulation by alcohol overconsumption—especially of leptin (which decreases bone mass through the central nervous system) and vitamin D/parathormone (which may interfere with bone metabolism) [51]. Moreover, excessive alcohol intake may impair nutrient absorption, including malabsorption of calcium from the intestine, leading to decreased levels of circulating serum calcium and calcium deficiency [52]. There are no univocal results of difference in the effects of alcohol between sexes; chronic heavy drinking may in fact cause hormone deficiencies in both men and women. Men with alcoholism may produce less testosterone—a hormone linked to the production of osteoblasts (cells that stimulate bone formation) [53]. In women, overconsumption of alcohol can decrease estrogens, which are generally low after menopause; lack of estrogen stimulation can lead to bone loss due to accelerated bone turnover in favor of resorption rather than formation [54]. The observed contrasting results reported in this meta-analysis may depend on the different age ranges included in the original studies, since the risk of osteoporosis and detrimental effects of alcohol on bone health spike after menopause [51]. Moreover, some studies have reported that women may have a higher sensitivity to alcohol and, therefore, their upper limit for moderate consumption might be slightly lower than for men [55]. Finally, alcohol may indirectly play a role in individuals’ BMI, as well as their lean and fat mass—which, in turn, has been shown to differentially contribute to BMD in men and women (stronger relationship in men than in premenopausal women) [56].

In line with the findings reported in this study, some other mechanistic studies support the hypothesis that moderate alcohol consumption may actually exert beneficial effects on bone health. Low-to-moderate alcohol intake has been associated with slowing age-related bone loss by decreasing the overall rate of bone remodeling [57]. The mechanism related to the alcohol content of alcoholic beverages may rely on the relative increase in estrogen levels in women and the increase in estradiol levels in men associated with moderate alcohol consumption [58]. Moreover, there is growing evidence that the non-alcoholic fraction of alcoholic beverages may play a role in improving bone health; for instance, red wine is rich in resveratrol—a stilbene with phytoestrogenic properties that may serve as a natural alternative to conventional hormone replacement therapy, exhibiting estrogen-like effects on bone metabolism [59]. Interestingly, resveratrol has also been demonstrated in animal studies to exert multiple actions on both osteoblasts and osteoclasts in the pre-menopausal period, albeit these findings are not found in humans [60]. Moreover, other phenolic compounds contained in beer—such as phenolic acids—have been linked to ROS scavenging activity and downregulation of inflammatory mediators, including osteoclast differentiation and activity by the osteoblast production of various signaling proteins [61]. Furthermore, silicon from malt contained in wine and, more importantly, in beer, has been reported to play a role in the growth and mineralization of bone and regeneration of connective tissue [62].

The present study has some limitations that should be taken into account when considering the results. First, the observational design of the original studies included does not allow us to assert causality of relationships, but only the association between consumption of alcohol and the outcomes investigated. Second, the alcohol intake was generally assessed during interviews or visits, and may not reflect early exposure or a lifelong habit. Third, the assessment of alcohol consumption fails to identify drinking patterns, which can only be hypothesized. Fourth, the increases in BMD values in light- compared to non-drinkers are not clinically relevant; however, these results should not be interpreted as a clinical improvement in BMD values in light drinkers, but rather as a null effect.

## 5. Conclusions

In conclusion, there is consistent evidence that increased alcohol consumption is associated with higher risk of osteoporotic fracture. However, the role of alcohol at lower doses is uncertain, given that BMD was even higher in moderate consumers compared to abstainers. The complexity of interpreting results on alcohol consumption summarizes the mixed interpretation of this major public health concern. There is confusion between modern alcohol drinking patterns and alcohol consumption within the context of a cultural heritage actually delivering health benefits associated with consolidated habits, such as a moderate daily consumption during meals—as opposed to binge drinking over a restricted time of the day. Therefore, alcohol consumers should consider that both the doses and patterns of consumption may impact bone health. Future studies are needed in order to elucidate the role of the behaviors related to alcohol consumption and their relationship with bone health.

## Figures and Tables

**Table 1:** Background characteristics of the studies included in the meta-analysis.

Author, Year	Design (Follow-Up)	Cohort (Country)	Sex and Sample Size	Age, Mean (Range)	Location of BMD Measurement/Fracture
**BMD Measurement**					
Felson, 1995	Cohort	FOSS (US)	M: 438, F: 691	75.9 (68–96) y	Proximal femur: neck, Trochanter, Ward’s triangle
Feskanich, 1999	Cross-sectional	NHS (US)	F: 188	63.0 (50–74) y	Femur neck, lumbar spine, hip
Ganry, 2000	Cross-sectional	EPIDOS (France)	F: 7598	79.9 (75+) y	Femur neck, Ward’s triangle, whole body
Cawthon, 2006	Cross-sectional	MrOS (US)	M: 5995	73.7 (65+) y	Femur neck, hip, lumbar spine
Mukamal, 2007	Cross-sectional	CHS (US)	MF: 5865	73.3 (65+) y	Femur neck, hip
Wosje, 2007	Cross-sectional	NHANES III (US)	MF: 13,512	48.0 (20+) y	Femur neck, hip
Tucker, 2009	Cohort	FOS (US)	M: 1182, F: 1537	61.5 (29–86) y	Femur neck, hip, spine
Kouda, 2011	Cross-sectional	FORMEN (Japan)	M: 1421	73.9 (65+) y	Femur neck, hip, spine
McLernon, 2012	Cross-sectional	APOSS (UK)	F: 3218	55.0 (50–62) y	Femur neck, lumbar spine
Coulson, 2013	Cross-sectional	GOS (Australia)	M: 534	79.0 (65+) y	Femur neck, spine, whole body
Cho, 2018	Cross-sectional	KNHANES IV & V (South Korea)	M: 2657, F: 2080	63.8 (50–79) y	Femur neck, lumbar, whole body
**Risk of fractures**					
Felson, 1988	Cohort (22.5 y)	FHS (US)	MF: 5209	NA (28–62) y	Hip (65)
Hernandez-Avila, 1991	Cohort (6 y)	NHS (US)	F: 88,484	NA (34–59) y	Forearm (593), hip (65)
Hemenway, 1994	Cohort (5.2 y)	HPFS (US)	M: 51,529	NA (40–75) y	Wrist (271), hip (67)
Hoidrup, 1999	Cohort (13.6 y)	CCPPS (Denmark)	M: 17,868, F: 13,917	50.5 (20–93) y	Hip (703)
Hansen, 2000	Cohort (6.5 y)	IWHS (US)	F: 34,703	NA (55–69) y	Total (4378), wrist (1128), forearm (288), upper arm (389), hip (275), vertebral (416)
Kanis, 2005	Cohort (8 y)	CaMos, DOES, Rotterdam Study (Netherlands, Australia, Canada)	M: 6036, F: 11,265	65.0 (25–103) y	Hip (279), spine, pelvis, ribs, distal forearm, and forearm (1207)
Cawthon, 2006	Cohort (3.6 y)	MrOS (US)	M: 5995	73.7 (65+) y	Hip (46), nonvertebral (256)
Mukamal, 2007	Cohort (12 y)	CHS (US)	MF: 5865	73.3 (65+) y	hip (84)

APOSS: Aberdeen Prospective Osteoporosis Screening Study; CaMos: Canadian Multicentre Osteoporosis Study; CCPPS: Copenhagen Centre for Prospective Population Studies; CHS: Cardiovascular Health Study; EPIDOS: Epidemiologie de l’Osteoporose Study; F: female; FHS: Framingham Heart Study; FORMEN: Fujiwara-kyo Osteoporosis Risk in Men; FOS: Framingham Offspring Study; FOSS: Framingham Osteoporosis Study; GOS: Geelong Osteoporosis Study; HPFS: Health Professionals Follow-Up Study; IWHS: Iowa Women’s Health Study; KNHANES: Korean National Health and Nutrition Examination Surveys; M: male; MrOS: Osteoporotic Fractures in Men Study; NA: not available; NHANES: National Health and Nutrition Examination Survey; NHS: Nurses’ Health Study; NHANES: National Health and Nutrition Examination Survey; y: years.

**Table 2:** Numerical data of the dose–response meta-analysis of the risk of hip and any fracture for various doses of alcohol consumption.

Site of Fracture	Datasets (Studies)	Nonlinear Analysis						
		Alcohol Consumption, Drinks/Day, RR (95% CI) *					*I*^2^ (%)	*P_heterog_*
		0	1	2	3	4		
**Hip**	8 (7)	1 (ref.)	0.99 (0.83; 1.19)	1.12 (0.88; 1.41)	1.33 (1.04; 1.69)	1.59 (1.23; 2.05)	4.2	0.405
**Any site**	8 (7)	1 (ref.)	1.03 (0.87; 1.21)	1.07 (0.95; 1.20)	1.13 (0.95; 1.36)	1.21 (0.83; 1.77)	26.8	0.161

## Supplementary Materials

The following supporting information can be downloaded at https://www.mdpi.com/article/10.3390/ijerph19031515/s1: Figure S1: Graphical representation of intercepts and slopes of the linear regression examining the relationship between alcohol consumption and age, BMI, and percentages of current smokers and physically inactive individuals; Figure S2: Funnel plots of risk of hip and any fracture for the highest vs. the lowest categories of alcohol consumption; Figure S3: Meta-analysis of risk of hip and any fracture for the highest vs. lowest categories of alcohol consumption by sex; Figure S4: Meta-analysis of risk of hip and any fracture for the highest vs. lowest categories of alcohol consumption by geographical region; Table S1: Numerical data of summarized mean differences in bone mineral density in different body segments between various doses of alcohol consumption vs. no consumption; Table S2: Summary slopes of the linear regression examining the relationship between alcohol consumption and age, BMI, and percentages of current smokers and physically inactive individuals; Table S3: Dose–response meta-analysis of risk of hip and any fracture for various doses of alcohol consumption by sex; Table S4: Dose–response meta-analysis of risk of hip and any fracture for various doses of alcohol consumption by geographical region.
