# Supplementary material for: Alcohol Consumption, Bone Mineral Density, and Risk of Osteoporotic Fractures: A Dose–Response Meta-Analysis
Source: Int J Environ Res Public Health. 2022 Jan 28;19(3):1515. doi: 10.3390/ijerph19031515 (PMC8835521; doi:10.3390/ijerph19031515)
Supplement: Supplementary file 1 [file ijerph-19-01515-s001.zip › ijerph-1559728-supplementary.pdf]

Supplementary Figure S1. Graphical representation of intercepts and slopes of the linear regression examining the relationship between alcohol consumption and age, BMI, percentage of current smokers and physically inactive individuals.

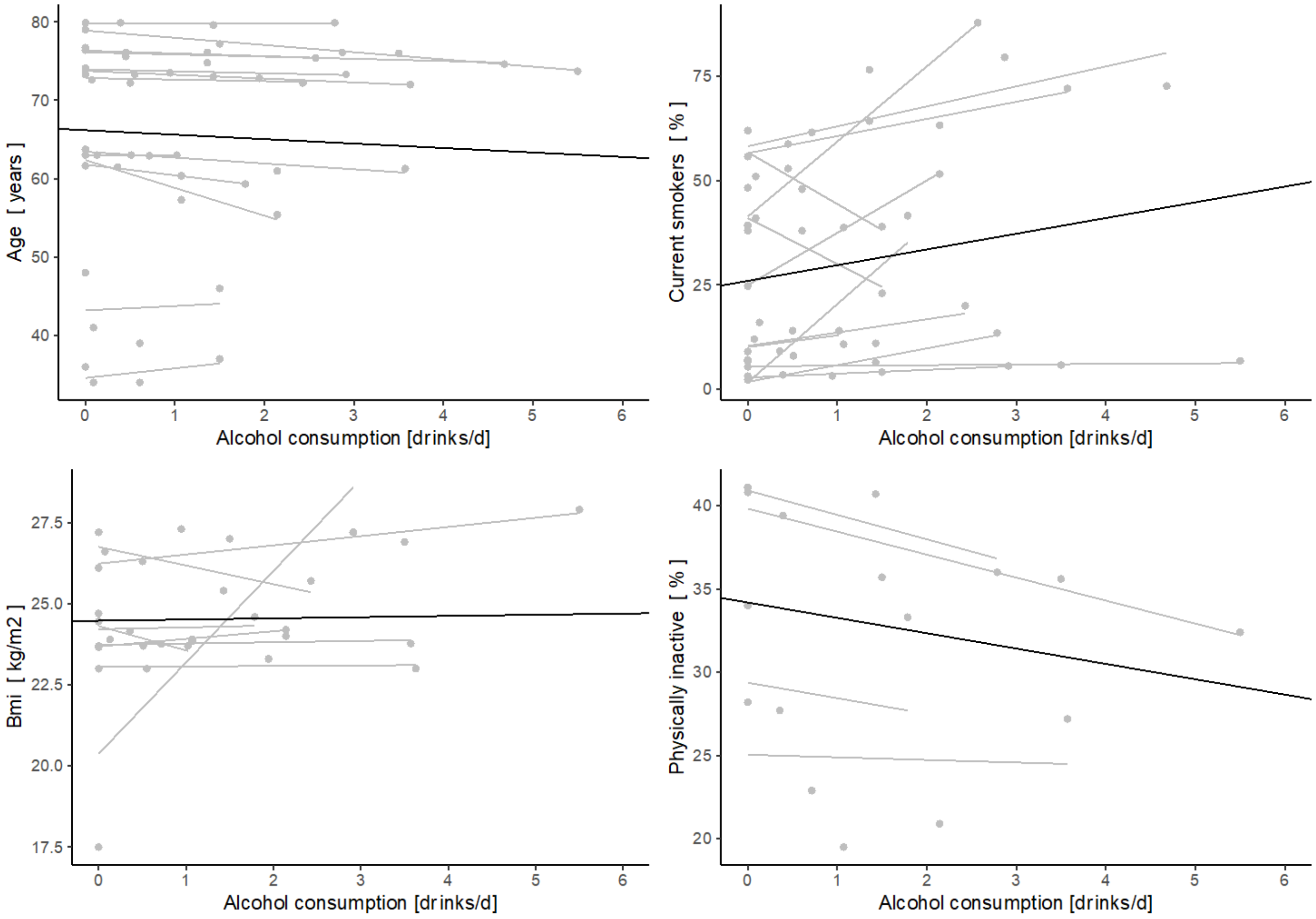

Supplementary Figure S2. Funnel plots of risk of hip and any fractures for the highest vs. the lowest category of alcohol consumption.

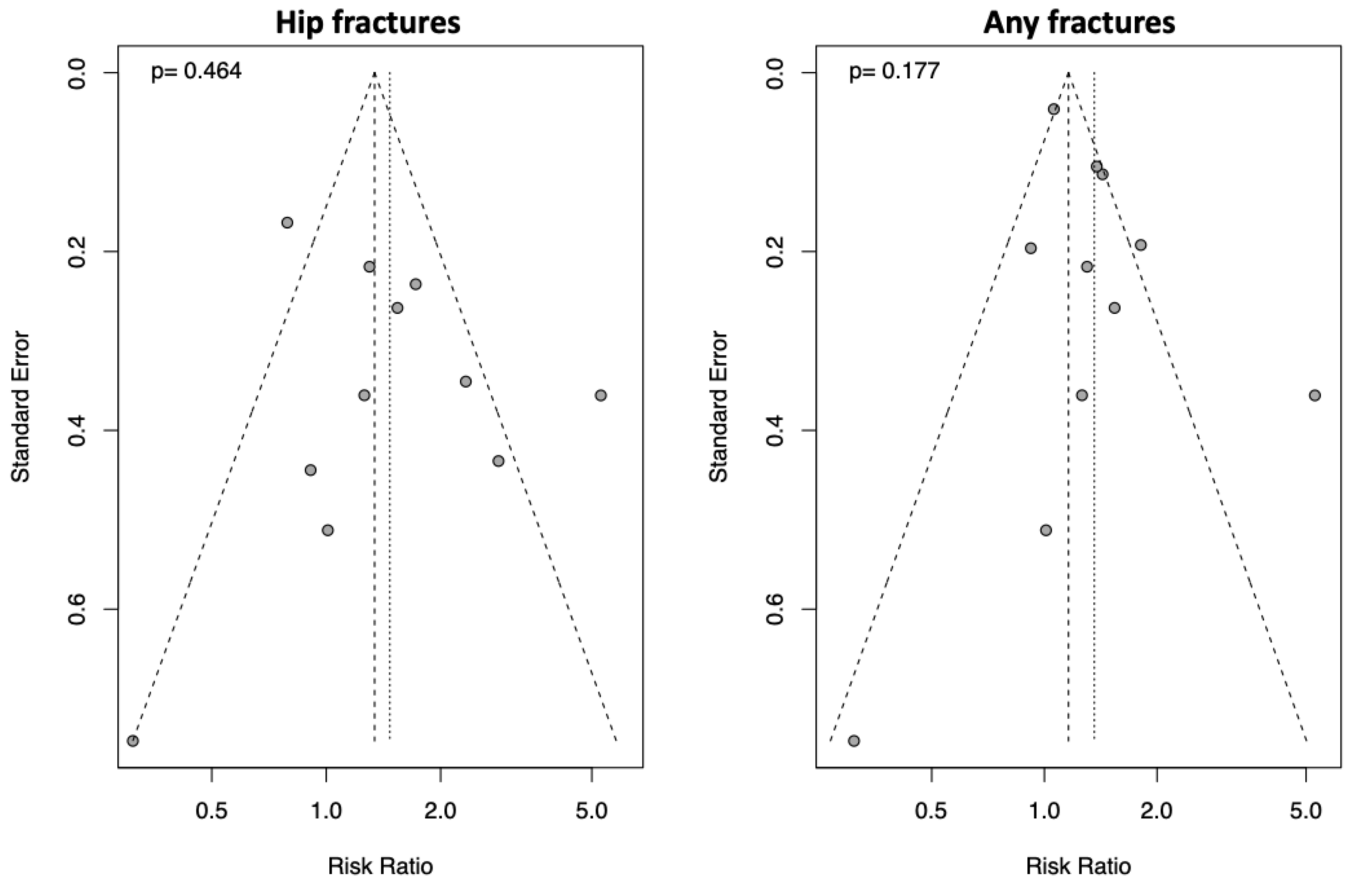

Supplementary Figure S3. Meta-analysis of risk of hip and any fracture for the highest vs. lowest category of alcohol consumption by sex.

Hip fractures by sex

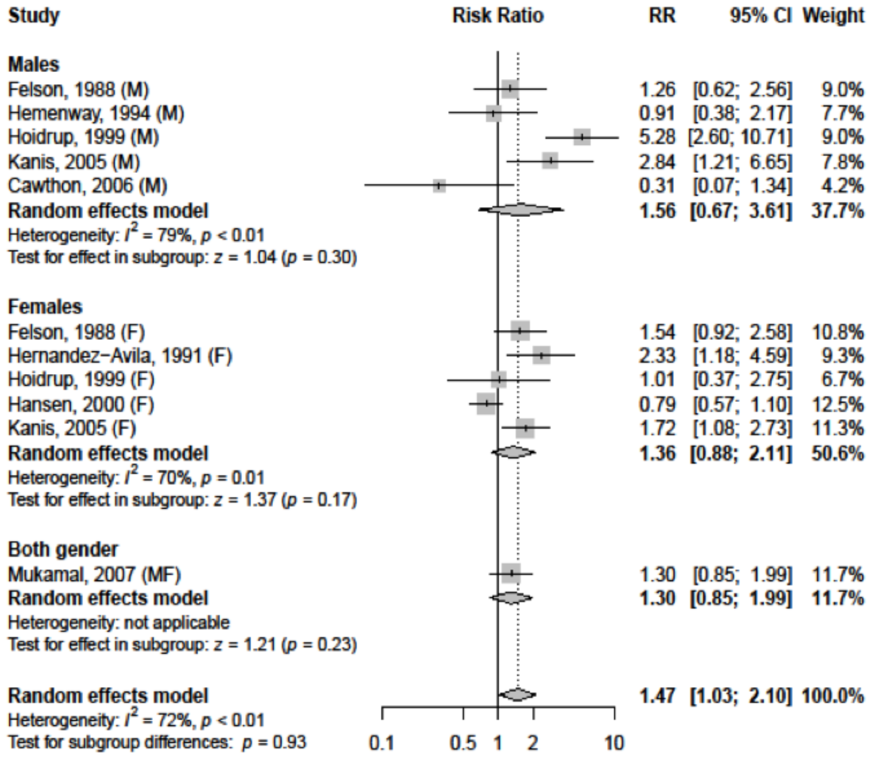

Any fractures by sex

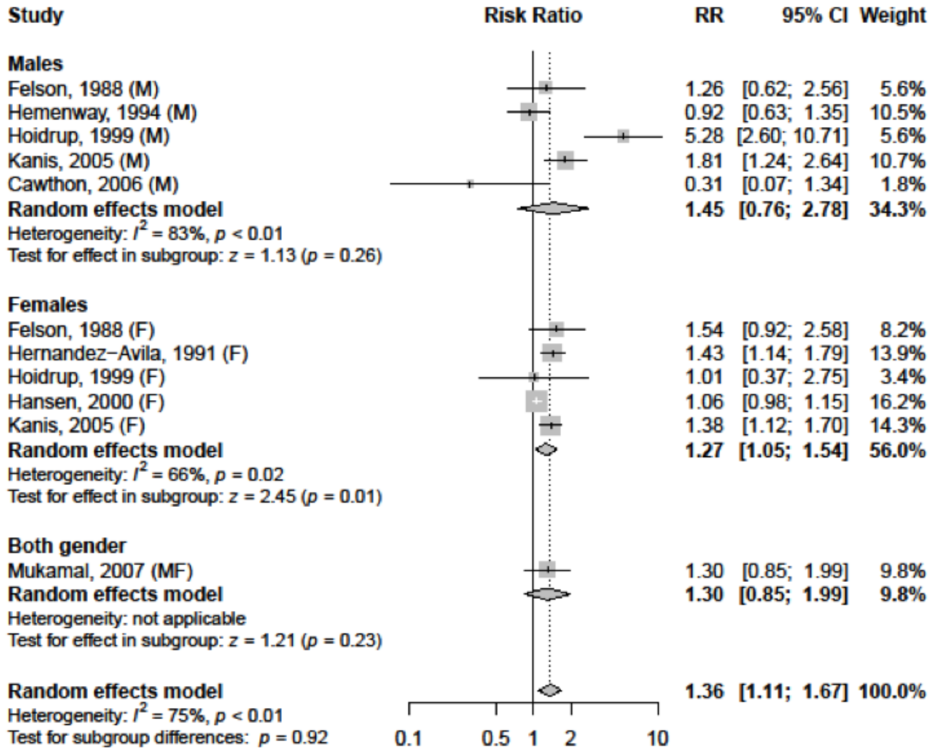

Supplementary Figure S4. Meta-analysis of risk of hip and any fracture for the highest vs. lowest category of alcohol consumption by geographical region.

Hip fractures by region

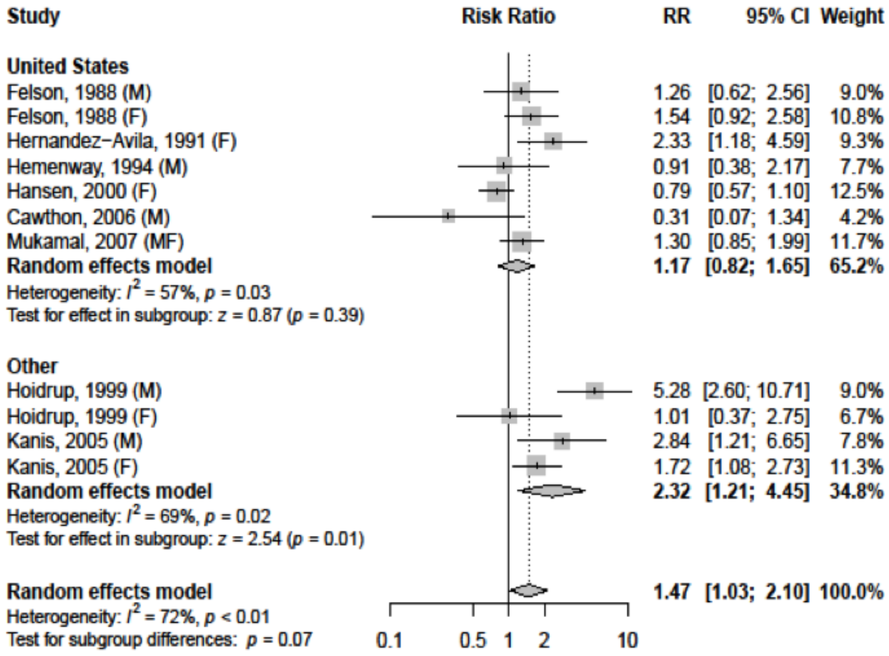

Any fractures by region

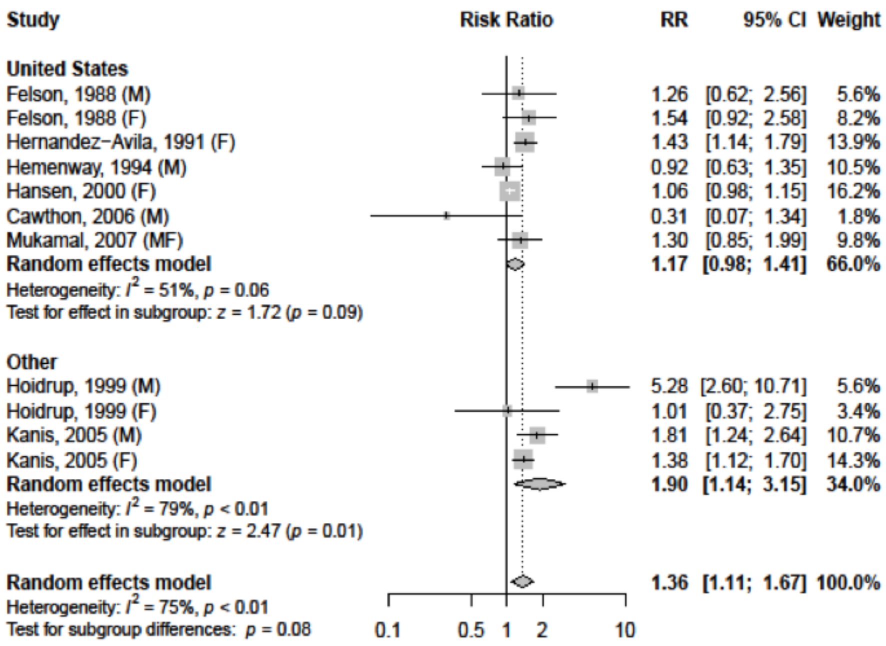

Supplementary Table S1. Numerical data of summary mean differences of bone mineral density in different body segments between various doses of alcohol consumption vs. no consumption.

**BMD**	**Datasets (studies)**	**Alcohol consumption, drinks/day, Mean difference [95% CI]**					***I^2[%]***	***$P_{heterog}$***
		**0**	**1**	**2**	**3**	**4**		
**Lumbar spine**	12 (8)	0 [ref.]	**0.02 [0.005; 0.035]**	**0.043 [0.002; 0.083]**	0.068 [-0.006; 0.141]	0.092 [-0.015; 0.2]	96.7	<0.001
**Femur neck**	16 (11)	0 [ref.]	**0.013 [0.005; 0.021]**	**0.016 [0.002; 0.030]**	0.013 [-0.026; 0.051]	0.009 [-0.056; 0.075]	99.9	<0.001
**Hip**	10 (7)	0 [ref.]	**0.018 [0.006; 0.03]**	0.015 [-0.007; 0.037]	0.005 [-0.047; 0.057]	-0.005 [-0.09; 0.08]	100	<0.001

Supplementary Table S2. Summary slopes of the linear regression examining the relationship between alcohol consumption and age, BMI, percentage of current smokers and physically inactive individuals.

Confounder	Both gender			Males			Females		
	Datasets (studies)	Slope (95% CI)	p	Datasets (studies)	Slope (95% CI)	p	Datasets (studies)	Slope (95% CI)	p
**Age [years]**	13 (10)	**-0.57 (-0.87; -0.26)**	**<0.001**	7 (7)	**-0.83 (-1.30; -0.37)**	**<0.001**	5 (5)	-0.32 (-0.97; 0.33)	0.337
**BMI [kg/m^2]**	8 (7)	0.04 (-0.17; 0.24)	0.728	5 (5)	**0.16 (0.02; 0.30)**	**0.027**	2 (2)	-0.25 (-1.09; 0.59)	0.563
**Current smokers [%]**	12 (9)	3.77 (-1.31; 8.85)	0.146	6 (6)	2.30 (-3.64; 8.25)	0.448	5 (5)	6.22 (-5.11; 17.54)	0.282
**Physically inactive [%]**	4 (3)	**-0.92 (-1.83; -0.01)**	**0.047**	2 (2)	-0.77 (-2.20; 0.66)	0.289	2 (2)	-1.22 (-2.74; 0.30)	0.116

Supplementary Table S3. Dose-response meta-analysis of risk of hip and any fracture for various doses of alcohol consumption by sex.

**BDM**	**Datasets (studies)**	**Alcohol consumption, drinks/day, Mean difference [95% CI]**						
		**0**	**1**	**2**	**3**	**4**	***I^2[%]***	***$P_{heterog}$***
**Lumbar**								
**Males**	6 (6)	0 [ref.]	**0.013 [0.010; 0.016]**	**0.020 [0.016; 0.025]**	**0.024 [0.013; 0.036]**	**0.028 [0.007; 0.048]**	59.8	0.006
**Females**	6 (5)	0 [ref.]	0.027 [-0.003; 0.056]	0.076 [-0.006; 0.158]	0.139 [-0.018; 0.295]	0.203 [-0.032; 0.438]	93.8	<0.001
**Femur neck**								
**Males**	7 (7)	0 [ref.]	**0.024 [0.017; 0.031]**	0.017 [-0.004; 0.039]	-0.005 [-0.071; 0.060]	-0.030 [-0.142; 0.082]	100	<0.001
**Females**	8 (7)	0 [ref.]	0.004 [-0.004; 0.013]	0.014 [-0.004; 0.032]	0.027 [-0.016; 0.068]	0.039 [-0.029; 0.108]	89.8	<0.001
**Hip**								
**Males**	4 (4)	0 [ref.]	**0.026 [0.015; 0.037]**	0.01 [-0.033; 0.052]	-0.024 [-0.135; 0.088]	-0.058 [-0.239; 0.124]	100	<0.001
**Females**	5 (4)	0 [ref.]	0.011 [-0.009; 0.031]	0.014 [-0.011; 0.038]	0.013 [-0.01; 0.036]	0.013 [-0.009; 0.034]	98	<0.001

Supplementary Table S4. Dose-response meta-analysis of risk of hip and any fracture for various doses of alcohol consumption by geographical region.

**BDM**	**Datasets (studies)**	**Alcohol consumption, drinks/day, Mean difference [95% CI]**						
		**0**	**1**	**2**	**3**	**4**	***I^2[%]***	***$P_{heterog}$***
**Lumbar**								
**US**	7 (4)	0 [ref.]	0.017 [-0.014; 0.047]	0.054 [-0.02; 0.127]	0.102 [-0.028; 0.233]	0.153 [-0.038; 0.344]	96.6	<0.001
**Other**	5 (4)	0 [ref.]	**0.015 [0.009; 0.022]**	**0.021 [0.019; 0.023]**	**0.022 [0.009; 0.035]**	0.021 [-0.006; 0.048]	39.6	0.104
**Femur neck**								
**US**	10 (6)	0 [ref.]	**0.014 [0.004; 0.024]**	0.011 [-0.008; 0.03]	-0.001 [-0.056; 0.054]	-0.014 [-0.109; 0.081]	99.9	<0.001
**Other**	6 (5)	0 [ref.]	**0.012 [0.000; 0.023]**	**0.017 [0.003; 0.031]**	**0.019 [0.005; 0.034]**	**0.021 [0.004; 0.038]**	93.7	<0.001
**Hip**								
**US**	8 (5)	0 [ref.]	**0.018 [0.005; 0.032]**	0.015 [-0.012; 0.042]	0.004 [-0.064; 0.071]	-0.008 [-0.118; 0.102]	99.9	<0.001
**Other**	2 (2)	0 [ref.]	0.014 [-0.02; 0.048]	0.018 [-0.027; 0.062]	0.018 [-0.029; 0.064]	0.018 [-0.03; 0.065]	98.5	<0.001
